# Impact of instrumentation material on local recurrence: a case-matched series using carbon fiber-PEEK vs. titanium

**DOI:** 10.1007/s11060-024-04842-9

**Published:** 2024-10-04

**Authors:** Jacob Ward, Mark Damante, Seth Wilson, Vicente Coelho, Dominic Franceschelli, Ahmed Nader Elguindy, Evan M. Thomas, Simeng Zhu, Dukagjin Blakaj, Sasha Beyer, Raju Raval, Raj Singh, David S. Xu, J. Bradley Elder, Joshua D. Palmer, Vikram B. Chakravarthy

**Affiliations:** 1 The Ohio State University College of Medicine, Columbus, USA; 2 Department of Neurological Surgery, The Ohio State University Wexner Medical Center, Columbus, USA; 3 Department of Radiation Oncology, The Ohio State University Wexner Medical Center, Columbus, USA

**Keywords:** Spine oncology, Carbon fiber reinforced peek, Titanium, Stereotactic body radiotherapy, Hardware failure

## Abstract

**Purpose:**

Spine metastases are a major burden of oncologic care, contributing to substantial morbidity. A well-established treatment paradigm for patients with metastatic epidural spinal cord compression includes separation surgery followed by stereotactic body radiotherapy (SBRT). Innovations in implant technology have brought about the incorporation of Carbon fiber-reinforced polyetheretherketone (CFR-PEEK) instrumentation for spinal fixation. We present our experience of CFR-PEEK instrumentation, comparing outcomes and complication profiles with a matched cohort of titanium instrumented cases for spine metastatic disease.

**Methods:**

Oncology patients who underwent spinal fusion for metastatic spine disease from 2012 to 2023 were retrospectively reviewed. Ninety-nine cases with CFR-PEEK fusions were case-control matched with 50 titanium controls (2:1 ratio) based upon primary tumor type and spinal instability neoplastic score (SINS) location. Demographic, clinical, radiographic and progression free survival (PFS) were analyzed.

**Results:**

In the study years, 263 patients underwent spinal decompression and fusion, for which 148 patients met predetermined inclusion criteria. Of these, 49 had titanium instrumentation, and 99 had CFR-PEEK. Complication profiles, including hardware failure and infection were similar between the groups. There was no significant difference in PFS between all CFR-PEEK and titanium patients (143 days versus 214 days; *p* = 0.41). When comparing patients in which recurrence was noted, CFR-PEEK patients had recurrence detected two times earlier than titanium patients (94 days versus 189 days; *p* = 0.013).

**Conclusion:**

In this case matched cohort, CFR-PEEK demonstrated decreased overall PFS suggestive of earlier local recurrence identification. Long-term studies are warranted for better evaluation of the impact on survival and systemic disease progression.

## Introduction

Spine metastases are a major source of oncologic morbidity, leading to significant pain and neurologic dysfunction [1–3]. As the field of spine oncology continues to evolve, the management of metastatic and primary tumors of the spine has become more individualized where decisions regarding chemotherapy, surgery, and/or radiation are based on a multitude of factors [4]. Surgery for epidural spinal cord compression was once viewed as palliative for preservation of neurologic function and pain control. However, it has become therapeutic for many patients with clinical trial data demonstrating a significant impact on patient survival, particularly with advances in radiotherapy. While effective, the first large series evaluating instrumentation failure in surgery for metastatic spine disease was by Amankulor et al. in a cohort of 318 patients. They demonstrated a hardware failure rate of 2.8%, however a key risk factor in developing symptomatic hardware failure was a construct length greater than six contiguous levels [5]. Stereotactic body radiotherapy (SBRT) compared to conventional post-operative radiation therapy (PORT) has shown to provide excellent local tumor control for both primary and metastatic tumors, particularly for those traditionally considered to be radioresistant [6–8]. High-quality postoperative imaging is crucial in accurate tumor contouring for radiation treatment to maximize local control and optimize disease surveillance.

Traditional titanium-based surgical constructs produce significant imaging artifact, particularly in the lateral recess and epidural space, complicating both radiation planning and tumor surveillance [9]. Carbon fiber-reinforced polyetheretherketone (CFR-PEEK) is a composite material combining the strength and stiffness of carbon fiber with the biocompatibility and resistance to corrosion of PEEK. It has been used in various medical and surgical applications, including as an alternative to traditional titanium instrumentation in spine surgery [10]. Reduced MRI artifact from CFR-PEEK hardware allows for more precise contouring of the spinal cord and organs at-risk for patients requiring adjuvant therapy and may allow for increased adoption of SBRT [11]. CFR-PEEK has been shown to be both safe and feasible with proposed benefits of early tumor detection around the pedicle, lateral recess and adjacent neural foramen [12]. This study presents a case-control matched analysis evaluating oncologic outcomes in patients recieveing CFR-PEEK instrumentation compared to titanium for spinal metastatic disease. We hypothesize the reduction in metal artifact on imaging provided by CFR-PEEK instrumentation provides two potential benefits, (1) improved surveillance of recurrent disease at the surgical site, and (2) improved ability to contour residual disease and neural elements during radiation planning. Should these hypotheses be true, there is a potential for improved oncologic outcomes, particularly with respect to progression free survival at the operative level.

## Methods

### Study design and inclusion criteria

The presented study is an Institutional Review Board approved (#2016C0023) retrospective cohort study of oncology patients undergoing spinal fusions by one of five spine surgeons from 2012 to 2023 at a single institution. Patients underwent surgery for pathological vertebral fractures, spinal cord compression, metastatic lesions to the spine, and pain palliation. Inclusion criteria for this study were: (1) patient age > 18 years, (2) CFR-PEEK or titanium instrumentation utilized for spinal fixation, and (3) availability of post-operative imaging to determine recurrence. Patients who had spinal decompression without instrumentation were excluded. Case-control matching by primary malignancy and spinal instability neoplastic score (SINS) location was performed in a 2:1 ratio of CFR-PEEK to titanium instrumentation to overcome potential type 2 error due to small CFR-PEEK sample size.

### Data collection

Patient demographic including sex, age, smoking status and pack-year history, and history of spine surgery at the index level, defined as the level in which tumor burden was noted and was the center of the fusion construct, were collected. Clinical characteristics including primary malignancy, pre-operative systemic therapy, pre- and post-operative Eastern Cooperative Oncology group (ECOG) performance status, SINS, and duration of follow-up were collected.

Surgical information, including operative time, hospital length of stay, cement augmentation of pedicle screws, estimated blood loss (EBL), and implant information (material, number implanted) were assessed. Post-operative metrics included (PORT) details (time to PORT, prescription dose, fractionation, target location), imaging modality used for radiation planning, and post-operative systemic therapy agent. Radiographic and clinical follow-up included magnetic resonance imaging (MRI) information (time since operation, recurrence information) and complication details (time to complication, revision needed, management of complication) were collected. The protocol for post-operative imaging surveillance was similar between the arms of the study which included MRIs every 3–4 months based on scheduling and patient availability. Local disease progression and tumor recurrence were identified using post-operative MRI. Mechanical complications were defined as any type of hardware failure (such as rod fracture and screw loosening) and were assessed via follow-up imaging and post-operative visits.

Oncologic outcomes included overall survival (OS, defined as time from initial surgery to death), and progression free survival (PFS, defined as time from initial surgery to first radiographic progression at the index level). OS was analyzed between the CFR-PEEK and titanium cohort. PFS was analyzed first within the entire cohort at the index level. We then performed a sub-analysis among patients known to has disease progression at the index level to mitigate potential competing risk of death, or shorter median follow up time in the CFRP group, in the PFS analysis. Patients who were lost to follow-up were censored in median OS and PFS calculations, as were those who died prior to local disease recurrence in median PFS calculation.

### Statistical analysis

Continuous variables, normality was tested using a Shapiro-Wilk test. Parametric variables were presented as mean (± standard deviation), and non-parametric variables were presented as median (± IQR). Continuous variables were compared with independent-sample T-test (parametric) or Mann-Whitney U (nonparametric), and categorical variables compared with chi-square test, or Fisher exact test if fewer than five expected outcomes. The primary endpoints of the study were median overall survival (OS), and recurrence as measured by progression free survival (PFS). Both OS and PFS were compared with Kaplan-Meier curves and tested with Log-rank test. All statistical analyses were performed using the IBM SPSS Statistics (formal citation). A p-value of less than 0.05 was regarded as significant.

## Results

Between the study years, 263 patients underwent spinal decompression and fusion, for which 148 patients met predetermined inclusion criteria. After 2:1 case-controlled matching, 99 were treated with CFR-PEEK instrumentation and 49 were treated with titanium instrumentation.

Demographic data for the two groups is reported in Table 1. Median age (*p* = 0.47) and sex (*p* = 0.343) were not significantly different between groups. The incidence of previous spine surgery and radiotherapy between groups did not differ (*p* = 0.236 and *p* = 0.829, respectively). There was no significant difference in the distribution of smoking status between groups at time of initial surgery, nor in number the pack-years in the patients who either currently smoked or had a history of smoking. The median follow-up time was significantly longer for the titanium group compared to CFR-PEEK (236 days versus 122.5 days, *p* < 0.0005). The overall top three primary malignancies between all patients were renal (*n* = 20), prostate (*n* = 19), and poorly differentiated carcinoma (*n* = 14).

**Table 1:** Demographic data from the CFR-PEEK and Titanium cohorts

	CFR-PEEK	Titanium	*p*-value
Number of patients	99	49	
Average Age on DOS (year)	63.4	63.23	0.47^a^
Smoking			0.7103^b^
Current	14	9	
Former	43	22	
Never	42	18	
Median Pack years	20	25	0.12^c^
Sex (%M)	66.70%	75.5%	0.343^b^
Previous spine surgery	8	7	0.236^b^
Previous spine RT	21	9	0.829^b^
Median follow-up (days)	122.5	236	< 0.0005^c^

*a denotes T-test used for statistical analysis

*b denotes Fisher Exact test used for statistical analysis

*c denotes Mann-Whitney U test used for statistical analysis

Table 2 compares surgical characteristics and postoperative course, as well as oncologic outcomes between groups. The median number of index levels was 1 and 3 for CFR-PEEK and titanium, respectively (*p* < 0.001), and the length of construct spanned 5 levels for CFR-PEEK and 6 levels for titanium, respectively (*p* < 0.001). Patients who received CFR-PEEK instrumentation also had shorter operative time (*p* < 0.001), shorter hospital length of stay (*p* < 0.001) and decreased estimated blood loss (*p* < 0.001).

**Table 2:** Surgical characteristics, outcomes, and progression-free survival of the two cohorts

	CFR-PEEK	Titanium	*p*-value
Median number of index levels	1	3	< 0.001^a^
Median Length of surgery(min)	215	317	< 0.001^a^
Median Length of stay(days)	7	14	< 0.001^a^
Median # of levels constructed	5	6	< 0.001^a^
Cement augmented screws (%Y)	57%	4.1%	< 0.0005^b^
Median EBL(mL)	600	1600	< 0.001^a^
# of recurrences/progressions	21	15	0.22^b^

*a denotes Mann-Whitney U test used for statistical analysis

*b denotes Fishers Exact test used for statistical analysis

Table 3 describes adjuvant therapies administered to patients, as well as data evaluating functional status both pre- and post-operatively via ECOG scores. The percentage of patients receiving pre-operative systemic therapy was 32.3% for the CFR-PEEK group compared to 61.2% for the titanium group (*p* < 0.0005). Post operative systemic therapy was administered to 77.8% of CFR-PEEK patients compared to 46.9% of titanium patients (*p* < 0.0005). Utilization of PORT was similar between the two groups at 59.6% and 55.1% for CFR-PEEK and titanium (*p* = 0.6) respectively. Within the groups, PORT was performed with SBRT for 45 (76.3%) CFR-PEEK patients, and 15 (55.6%) titanium patients (*p* = 0.08). Of those treated with SBRT, the median prescription dose was 27 Gy for both CFR-PEEK and titanium (*p* = 0.91). There was no difference in the percent of patients with a pre-operative ECOG score of 0 and 1 between cohorts at 53% and 49% for CFR-PEEK and titanium, respectively (*p* = 0.73), as well as no significant difference in the percent of patients with a post-operative ECOG score of 0 and 1 at 32% for CFR-PEEK and 30.6% of titanium patients (*p* = 0.99).

**Table 3:** Adjuvant therapies and post-operative outcome measures of the two cohorts

	CFR-PEEK	Titanium	*p*-value
Received pre-op systemic therapy (%)	32.3%	61.2%	< 0.0005^a^
Received post-op systemic therapy (%)	77.8%	46.9%	< 0.0005^a^
Received post op RT(%)	59.6%	55.1%	0.6^a^
SBRT PORT (%)	45 (76.3%)	15 (55.6%)	0.08^a^
EBRT PORT (%)	14 (23.7%)	12 (44.4%)	0.08^a^
Median dose of post-op RT(cGy)	2700	2700	0.91^b^
% of patients with ECOG 0–1 Pre-op	0.53	0.48	0.73^a^
% of patients with ECOG 0–1 Post-op	32%	30.60%	1^a^

*a denotes Fisher Exact test used for statistical analysis

*b denotes Mann-Whitney U test used for statistical analysis

Table 4 describes data related to post operative complications and patient outcomes. There was no significant difference in any of the measured post operative complications, including hardware failure, infection, or reoperation for tumor recurrence. The CFR-PEEK cohort had one patient with post-operative hardware failure requiring subsequent extension of the fusion construct and the titanium group had none (*p* = 0.5511). The CFR-PEEK cohort had two patients with post operative infections and the titanium group had one. In terms of reoperation for tumor recurrence at the index level, the CFR-PEEK cohort had 3 patients and the titanium group had 1. The CFR-PEEK cohort had 11 patients with post operative complications unrelated to spine surgery as listed in Table 4 and the titanium group had 5. Complications in the “other” category included pulmonary embolism, pneumonia, pleural effusion, acute kidney injury, decubitus ulcers, urosepsis, new onset eye dysfunction, hematuria, and deep vein thrombosis.

**Table 4:** Post-operative complications of the two cohorts

	CFR-PEEK	Titanium	*p*-value
Post-op hardware failure	1	0	0.5511
Post-op infection	2	1	1
Post op tumor debulking	3	1	1
Other post-op complications not related to spine*	11	3	0.39

*Other complications included pulmonary embolism, pneumonia, pleural effusion, acute kidney injury, decubitus ulcers, urosepsis, new onset eye dysfunction, hematuria, and deep vein thrombosis

**Fig. 1:**
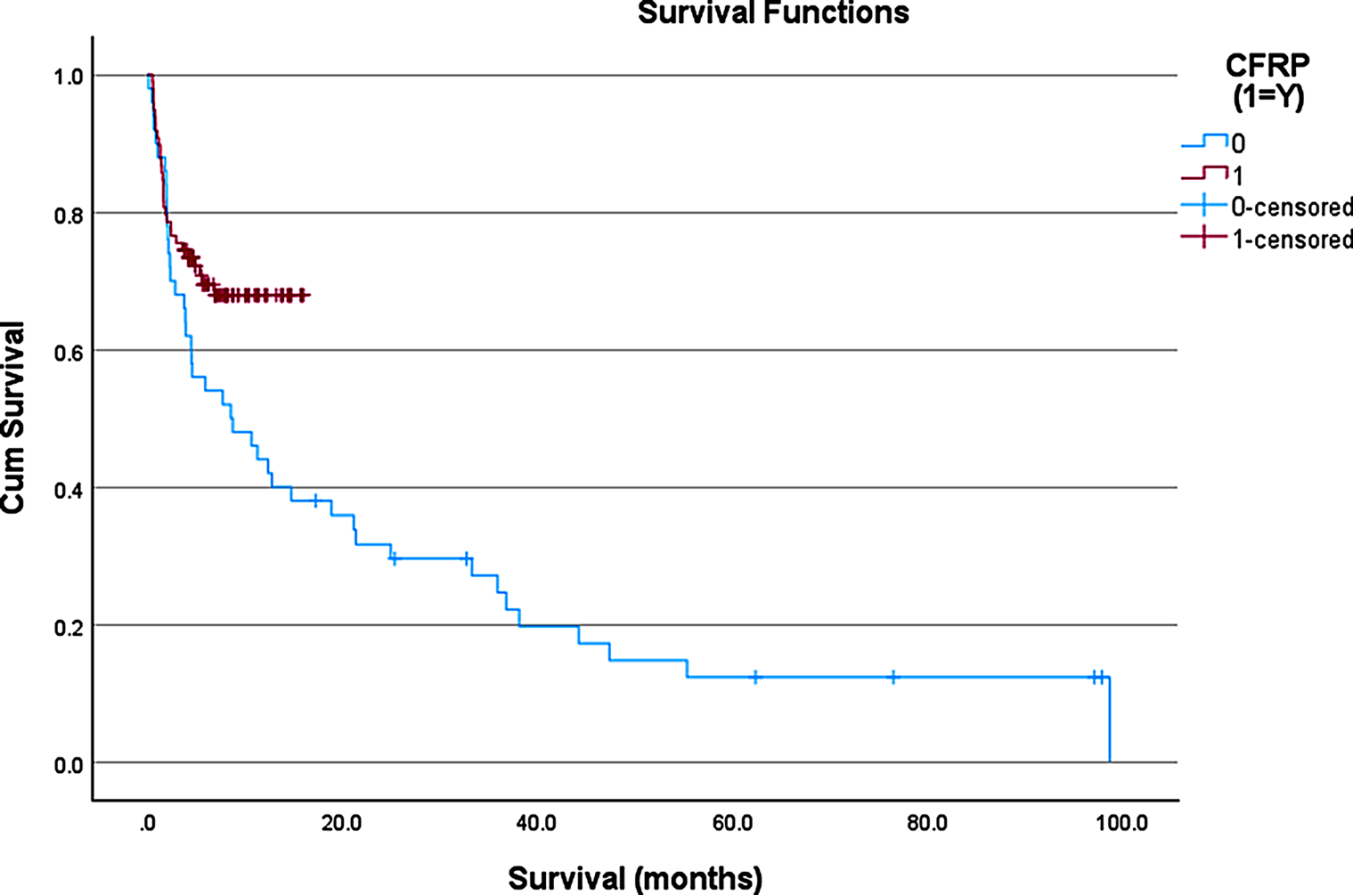
Kaplan-Meier curve representing the overall survival (OS) of patients with CFR-PEEK (red) and Titanium (blue) instrumentation. Censored patients represent patients alive at that time point but were either lost to follow up, or that time point was their most recent follow up based on surgical date

**Fig. 2:**
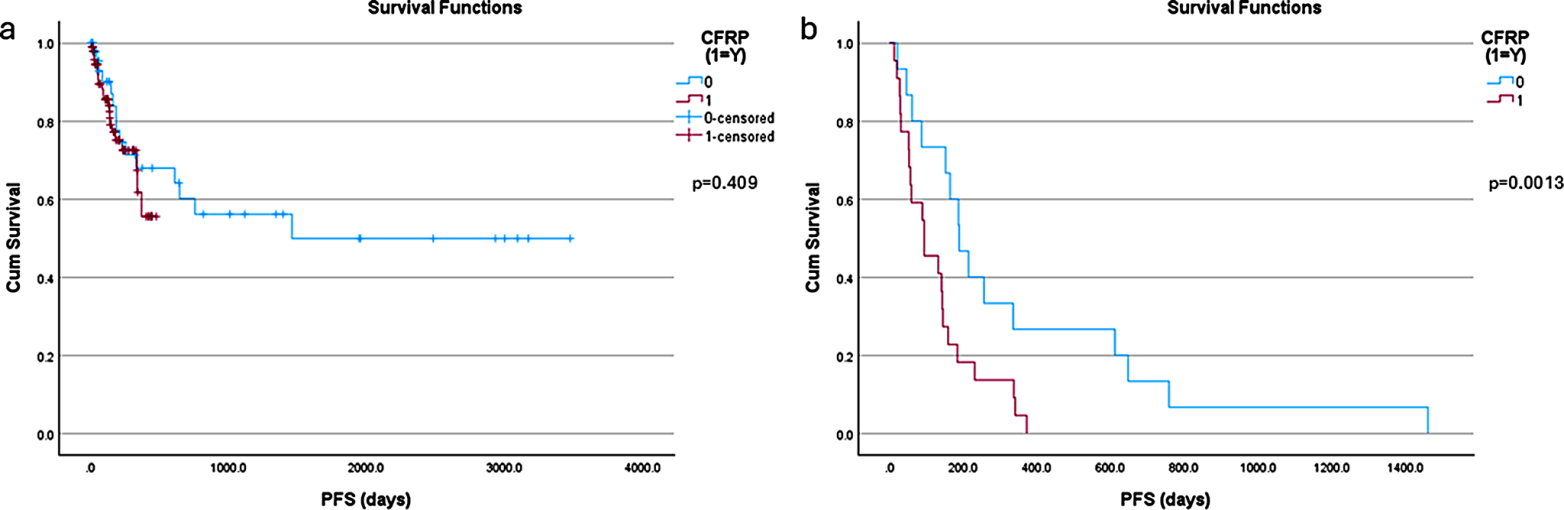
Kaplan-Meier curves representing the progression-free survival (PFS) of patients with CFR-PEEK (red) and Titanium (blue) instrumentation. (**a**) is the curves representing all patients from both cohorts (**b**) is the sub analysis curves for only patients in which recurrence/progression was noted at the index level, in each cohort. Censored patients represent patients alive at that time point but were either lost to follow up, or that time point was their most recent follow up based on surgical date

With respect to OS, the CFR-PEEK group had yet to reach the median OS compared to a median OS of 8.5 months in the titanium cohort (Fig. 1, p*-value = 0.011).* There was no significant difference in the incidence of local recurrence observed between the two groups at 22 (22.2%) for CFR-PEEK and 15 (30.6%) for titanium (*p* = 0.31). The PFS for all patients in the cohort was 143 days for the CFR-PEEK group and 214 days for the titanium group, respectively (*p* = 0.409, Fig. 2A). Among the patients that demonstrated recurrence, the recurrence was detected sooner in the CFR-PEEK group compared to the titanium group (94 days versus 189 days, respectively; *p* = 0.0013; Fig. 2B).

**Fig. 3:**
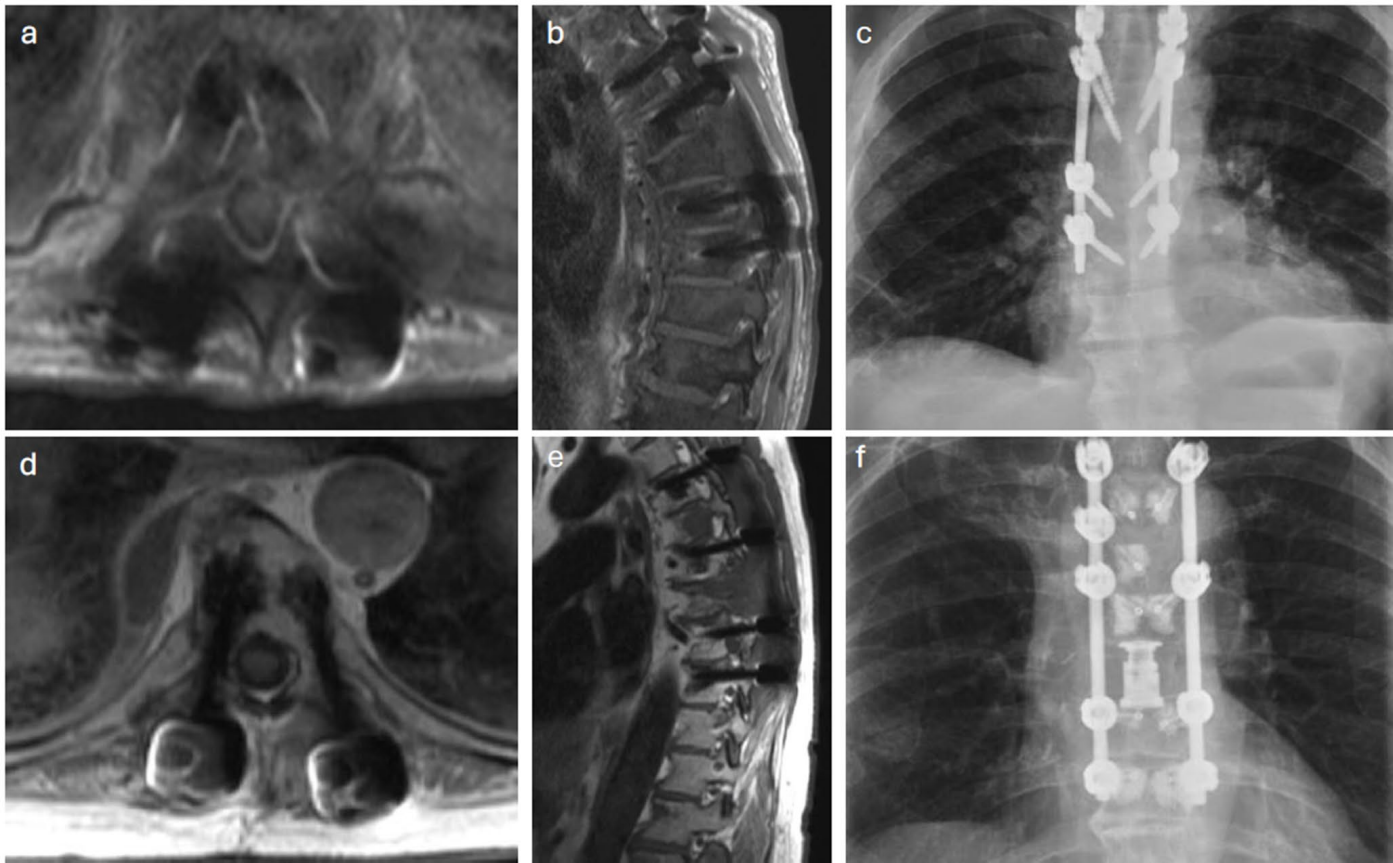
Images of local recurrence detection in patients with Titanium instrumentation (**a**-**c**), and CFR-PEEK instrumentation (**d**-**f**)

Figure 3 depicts the detection of tumor recurrence in one CFR-PEEK instrumented patient, and one titanium instrumented patient to highlight the differences in visualization of surrounding structures with the two materials.

## Discussion

The utilization of CFR-PEEK instrumentation allows for more precise contouring in SBRT planning and early detection of recurrent or progressive disease on surveillance imaging [13]. To date, most data of CFR-PEEK instrumentation describe institutional experience, including rates of hardware failure, with or without comparison to titanium instrumentation [9, 14–15]. Many of these studies have commented on the theoretical benefit of early recurrence detection due to diminished artifact on MRI, but none have tested the hypothesis. The presented study represents the first case-control matched analysis of CFR-PEEK versus titanium instrumentation for spine oncology patients to assess impact on oncologic outcomes, particularly early detection of recurrent or progressive disease.

Titanium instrumentation limits visualization of the ventral epidural space, particularly the region medial to the pedicle (often referred to as the lateral recess) and within the instrumented pedicle and vertebral body, regardless of imaging modality [16]. CFR-PEEK instrumentation has demonstrated improved post-operative visualization of the index levels when monitoring for local tumor recurrence and disease progression due to its reduced imaging artifact, as well as less dose perturbation and improvement in proton planning [17–19]. Poel et al. reinforced this notion, reducing image artifact by up to 90% compared to titanium in phantom models [20]. As such, CFR-PEEK allows for more precise contouring of the spinal cord and organs at risk, promoting the use of SBRT [11, 17]. With the increasing use of CFR-PEEK in spine oncology, there has also been a shift in the surgical approach. The shift focuses on more focal surgical treatment with shorter construct lengths and more spinal decompression [21–22]. This could account for the difference in surgical characteristics between the two cohorts. These surgical factors do not impact the potential artifact of the instrumentation at the level of disease for tumor recurrence monitoring.

Improved SBRT contouring was hypothesized to improve local control compared to the titanium cohort, however PFS was not significantly different between groups. However, of the patients that demonstrated recurrence/progression, this was identified twice as early in the CFR-PEEK group compared to the titanium group. Earlier detection of local recurrence can facilitate modifications in systemic therapies or pursuance of salvage radiotherapy, while avoiding additional surgical interventions [23–24]. With a change in surgical approach, an argument could be made about earlier recurrence in the CFR-PEEK group being due to procedural differences. However, case-matching the two groups by primary tumor type allowed control of oncologic predilection for recurrence. Of the 21 patients in which tumor recurrence/progression was noted, only 3 required additional surgery for recurrence, whereas the remaining 18 were managed with additional radiation therapy and changes to systemic therapy. Median OS had not yet been reached in our CFR-PEEK cohort and is predicted to be significantly longer compared to the Titanium cohort. We hypothesize that early detection of recurrent/progressive disease at the index disease level will allow for alteration in systemic therapy early enough in the disease course to halt or prolong systemic progression. This is difficult to evaluate in a retrospective study, as other explanation does include advances in systemic therapies available to patients in the CFR-PEEK group, as they were treated more recently than patients in the titanium group.

Previous work has shown CFR-PEEK to exhibit a complication profile, functional recovery, and biomechanical integrity comparable to titanium instrumentation [9, 14–15, 25–27]. Cofano et al. demonstrated in a comparative study between 36 CFR-PEEK patients, and 42 titanium patients, had no significant difference in post-operative clinical complications or hardware failures [9]. Similarly, Joerger et al. showed in 321 patients with CFR-PEEK instrumentation for spine metastasis patients, revision surgery for post-operative complications was necessary in 17.1% of patients, with rare implant-related complications of screw loosening (2.2%) [15]. Our cohort demonstrates a similar complication profile between CFR-PEEK and titanium with no significant differences in hardware failure, infections, and reoperation for tumor recurrence as seen in previously published work [9, 12, 15].

## Limitations

The limitations of this study are inherent due to its retrospective nature and evolution of oncologic treatment within the duration of the study. The 99 CFR-PEEK patients represent the first patients at our institution to be treated with this instrumentation. However, the case matched titanium patients who came from a different cohort of patients, were treated in a different era of oncology care. The change in surgical management and radiation treatment between the time frames of the two cohorts could alter the post-operative course. Additionally, due to the retrospective aspect of the study, we could not control for post-operative systemic therapy usage and did not control for this in analysis of PFS and OS. Overall, the evolution of patient care in recent years could act as a secondary contributor to improved outcomes in patients recently diagnosed with metastatic spinal disease.

## Conclusion

The presented study is the first case-control matched analysis comparing CFR-PEEK instrumentation to traditional titanium instrumentation. While overall PFS was not different between groups, recurrence/progression was detected two times earlier when CFR-PEEK instrumentation was utilized. This allows for adjustment in systemic and/or radiotherapy treatment plans, while avoiding additional surgical intervention, as demonstrated in this cohort.

## Data Availability

No datasets were generated or analysed during the current study.
