# Correction to: Impact of instrumentation material on local recurrence: a case-matched series using carbon fiber-PEEK vs. titanium

**DOI:** 10.1007/s11060-024-04860-7

**Published:** 2024-11-08

**Authors:** Jacob Ward, Mark Damante, Seth Wilson, Vicente Coelho, Dominic Franceschelli, Ahmed Nader Elguindy, Evan M. Thomas, Simeng Zhu, Dukagjin Blakaj, Sasha Beyer, Raju Raval, Raj Singh, David S. Xu, J. Bradley Elder, Joshua D. Palmer, Vikram B. Chakravarthy

**Affiliations:** 1 The Ohio State University College of Medicine, Columbus, USA; 2 Department of Neurological Surgery, The Ohio State University Wexner Medical Center, Columbus, USA; 3 Department of Radiation Oncology, The Ohio State University Wexner Medical Center, Columbus, USA

**Correction to: Journal of Neuro-Oncology**

10.1007/s11060-024-04842-9

In the caption to Fig. 3 in this article, the CFR-PEEK and Titanium instrumentation were said to show in figure parts **a–c** and **d–f**, resp., whereas it should have been the other way around, as follows:

**Fig. 3** Images of local recurrence detection in patients with Titanium instrumentation (**a–c**), and CFR-PEEK instrumentation (**d–f**).

The original article has been corrected.

